# A Novel GBM Saliency Detection Model Using Multi-Channel MRI

**DOI:** 10.1371/journal.pone.0146388

**Published:** 2016-01-11

**Authors:** Subhashis Banerjee, Sushmita Mitra, B. Uma Shankar, Yoichi Hayashi

**Affiliations:** 1 Machine Intelligence Unit, Indian Statistical Institute, Kolkata, India; 2 Department of Computer Science, Meiji University, Kawasaki, Japan; University of Pécs Medical School, HUNGARY

## Abstract

The automatic computerized detection of regions of interest (ROI) is an important step in the process of medical image processing and analysis. The reasons are many, and include an increasing amount of available medical imaging data, existence of inter-observer and inter-scanner variability, and to improve the accuracy in automatic detection in order to assist doctors in diagnosing faster and on time. A novel algorithm, based on visual saliency, is developed here for the identification of tumor regions from MR images of the brain. The GBM saliency detection model is designed by taking cue from the concept of visual saliency in natural scenes. A visually salient region is typically rare in an image, and contains highly discriminating information, with attention getting immediately focused upon it. Although color is typically considered as the most important feature in a bottom-up saliency detection model, we circumvent this issue in the inherently gray scale MR framework. We develop a novel pseudo-coloring scheme, based on the three MRI sequences, *viz*. *FLAIR*, *T*2 and *T*1*C* (contrast enhanced with Gadolinium). A bottom-up strategy, based on a new pseudo-color distance and spatial distance between image patches, is defined for highlighting the salient regions in the image. This multi-channel representation of the image and saliency detection model help in automatically and quickly isolating the tumor region, for subsequent delineation, as is necessary in medical diagnosis. The effectiveness of the proposed model is evaluated on MRI of 80 subjects from the *BRATS* database in terms of the saliency map values. Using ground truth of the tumor regions for both high- and low- grade gliomas, the results are compared with four highly referred saliency detection models from literature. In all cases the *AUC* scores from the *ROC* analysis are found to be more than 0.999 ± 0.001 over different tumor grades, sizes and positions.

## Introduction

Cancer has become the deadliest killer, worldwide, over the last decade [1]. By the time physical manifestations become evident, in many cases metastasis has occurred. This results in failure of local tumor control and poor patient prognosis. Quantitative imaging [2], using magnetic resonance imaging (MRI), computed tomography (CT), positron emission tomography (PET), etc., is playing an important role in improved tumor management through noninvasive detection, diagnosis, treatment and prognosis. These days one needs to integrate diverse, multimodal information in a quantitative manner, to provide specific clinical prediction for helping clinicians in accurately estimating patient outcomes.

Of all lethal brain tumors Glioblastoma multiforme (GBM) is the most common. Typically it has poor prognosis because its diagnosis and treatment are still largely guided by immunohistochemistry and histopathology [3]. It becomes challenging in brain tumor patients to have repeated tumor biopsies. Therefore noninvasive techniques like imaging is playing an important tools for assessing glioma during the treatment. MRI provides high spatial resolution and can detect abnormalities at minute level of the brain in terms of both shape and volume. It is being routinely used in the clinical diagnosis and disease characterization followed by disease management, since it provides a superior contrast of soft tissue structures. It is also safe, as it does not involve any exposure to radiation.

Reconstruction and display of detailed 3D images of the brain is possible using magnetic resonance imaging (MRI) [1]. Due to its dependence on biologically variable parameters such as longitudinal relaxation time (*T*1), transverse relaxation time (*T*2), and proton density (*PD*), using different pulse sequences and modifying the imaging parameters variable image contrast can be achieved in MRI. It is known that none of these sequences, individually, are able to depict the entire extent of a malignancy. For example, Fluid-Attenuated Inversion Recovery (*FLAIR*) causes damping of the ventricular CSF signal such that it appears dark. Different types of contrast enhancing agents, like Gadolinium (Gd), help in highlighting their pathological intra-tumoral take-up in *T*1-weighted MRI scans (*T*1*C*).

Segmentation and detection, of different regions of interest (ROI) in medical images, is manually performed by experts for treatment planning and diagnosis. Automated medical image analysis promises to play an important role in this scenario, particularly in overcoming human bias and the enormity of available data.

Tumors can exhibit different characteristics in different patients inspite of having originated in the same organ. Moreover, variations within a single tumor can cause marked differences among its imaging features—like necrosis or contrast enhancement; being primarily caused by changes in blood flow (or perfusion). Upon superimposing multiple sequences of MR images having prominent glioma regions, it is observed that poorly perfused areas in *T*1*C* images exhibit regions of low (or high) water content on *T*2-weighted images along with mismatches between perfusion and diffusion in the *FLAIR* sequence. The cells that are likely to be resistant to therapy belong to those regions having poor perfusion and high cell density, and are of particular clinical interest [4].

This highlights the utility of superposing multiple channels of MR imaging, like contrast enhanced *T*1-, and *T*2-weighted, as well as *FLAIR* components, in identifying and extracting heterogeneous tumor region(s).

**Fig 1:**
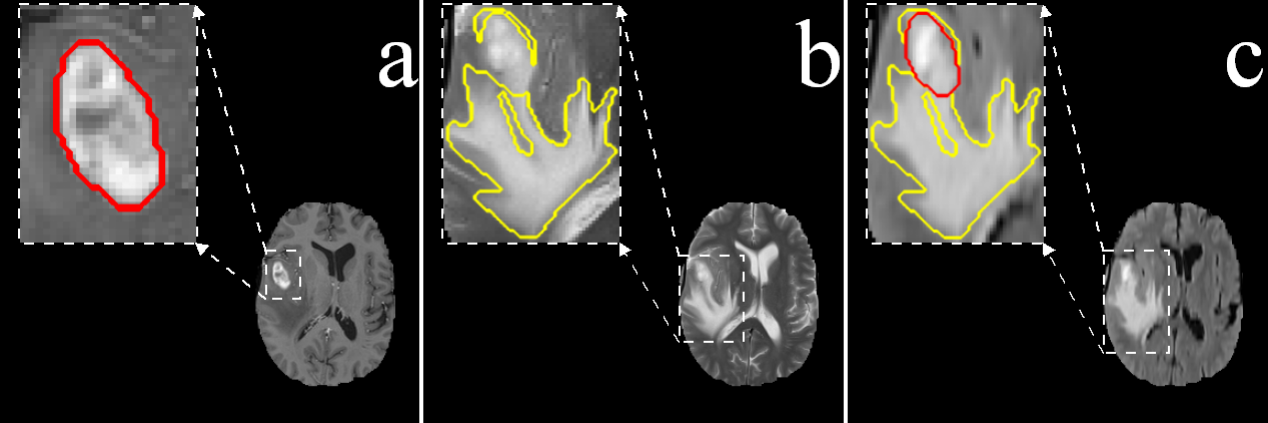
Primary MRI sequences of the brain. (a) *T*1*C* exhibiting enhanced tumor structure and boundary (Red), (b) *T*2 associated with edema or swelling (Yellow), and (c) *FLAIR* demonstrating both the edema (Yellow) and enhanced solid core (Red).

It is observed that radiologists typically delineate the gross tumor core from the T1C MR slices, because the tumor boundary becomes more visible due to emphasized contrast between gray and white matter (Fig 1(a)). The T2 channel, providing better contrast between brain tissue and cerebrospinal fluid (CSF), is preferred for delineating the edema region (Fig 1(b)). Although the edema boundary becomes fuzzy in FLAIR, both tumor and edema regions are appropriately visible here (Fig 1(c)). Each pixel in the tumor is, therefore, defined by its image intensity in different sequences, viz. (i) T1C, (ii) T2, and (iii) FLAIR.

The subjective assessment of an image depends heavily on identifying the salient region within it. The term “visual saliency” was coined by Ullman and Sha’ashua [5], and extended by Itti *et al*. [6] towards the development of a computational architecture. The human visual system is sensitive to the salient regions in an image, due to their high discriminative features, thereby resulting in early visual arousal. When we view a picture or a scene, our eyes immediately get drawn to the relevant (or salient) parts therein, on basis of the attention mechanism of the Human Visual System. The bottom-up visual attention is driven by the intrinsic low-level features of a scene. Top-down attention, on the other hand, is a high-level visual task requiring the search for a specific object. Considering an image as its input a computational saliency model typically generates a topographical map to determine the salient, attention-grabbing nature of each region from the perspective of a viewer in terms of human eye movement [7].

A perceptual quality of human vision, which grab the viewer’s attention and makes the object stand out from the rest is defined as visual saliency. It can also be defined as the outcome of comparing a central region with its surroundings, in terms of unpredictability, contrast and rarity [8, 9]. Saliency detection methods can be broadly classified into (i) biology-based [6, 10], (ii) fully computational [11, 12] and (iii) hybrid [13, 14] methods. The algorithms that detect saliency by using only low-level features, like color, intensity, orientation, incorporate the bottom-up strategy. Those in the top-down strategy include some learning from the training data involving the position or shape of a salient object. It has been observed that often attention is immediately drawn to a salient item, in spite of the existence of many other items (or distractors), without any need to scan the image. A visually salient region is typically rare in an image, and contains highly discriminating information. This concept can, therefore, be expected to have a major bearing towards the fast identification of an ROI or tumor from a medical image.

Existing literature on computational visual saliency models mainly deal with detection from the natural scenes. The ultimate aim of this research, on the other hand, is to develop a saliency-based framework for fast and automated detection of the whole tumor from multi-channel brain MRI involving glioma. Abnormality detection in medical imaging is a key step adopted by radiologists, as they manually search for lesions and/or other such abnormalities in the affected organ for the purpose of diagnosing and writing their report. In this context Computer-Aided Detection (CADe) plays an important role in assisting doctors and radiologists for interpreting medical images, and identifying (any) lesions. Therefore, improving CADe systems in the field of computer vision is an active research area—with particular emphasis on medical imaging.

The role of visual attention mechanism, in the context of medical images, is being investigated in literature. The objective is to model the visual search strategies of experts while also assisting them in improving detection. Nodine and Kundel [15] introduced perception study in case of medical images. They collected the eye (or gaze) tracking data of radiologists, while observing chest X-ray images in presence of tumors, to develop a model for predicting the sequence of events from the time of viewing the X-ray image upto the diagnostic decision-making [16]. Perception study was extended in the context of brain CT images for detecting lesions. For abnormality detection in medical images Jampani *et al*. [17] investigated the relevance of computational saliency models. They applied three popular methods, extended from the natural scene framework, to obtain saliency maps for finding lesions from color retinal images and chest X-Ray images. The results were validated against ground truth by medical experts. Automated lesions detection from retinal images based on visual saliency was also studied [18, 19].

Over the decade several methods have been developed to automate tumor segmentation, using neural networks, support vector machines (SVM), atlas-based methods, and outlier detection [20]. However satisfactory results often require either a complex prior model or a large amount of training data, thereby restricting the range of application. In this scenario our algorithm presents an intuitive method, by integrating multi-channel MR sequences to generate a pseudo-colored image in order to swiftly detect brain abnormalities without any prior training or supervision phase. We follow the perception pattern of radiologists through saliency detection. The most powerful aspect of our methodology is that it can be implemented in real-time and is robust to changes in parameters; thereby making it applicable to a wide range of MRI data.

We propose a novel saliency detection model for brain glioma mapping, in MRI, taking cue from visual saliency concept in natural scenes. Since attention is immediately drawn to any salient item, there is no need to scan the entire image. The image can, therefore, be processed in parallel to orient visual attention towards the most salient location very fast. Initially visual saliency is employed to quickly identify the ROI. The contribution lies in extending the concept of visual saliency-based object detection (from natural images [8]) to the medical domain, in order to automatically and simultaneously identify ROIs like tumor(s) from images. We develop a bottom-up saliency detection model, where color is typically considered as the most important feature. However we cannot use this feature in case of the inherently gray scale MR images. Therefore we design a novel pseudo-coloring strategy for MRIs, involving a combination of the three sequences, to generate saliency maps that provide the saliency strength at every pixel. Finally a 3D saliency map can be generated, by repeating the above process over each of the 2D MR slices extracted from these three sequences.

## Material and Methods

### Ethics Statement

“Brain tumor image data used in this work were obtained from the MICCAI 2012 Challenge on Multimodal Brain Tumor Segmentation (http://www.imm.dtu.dk/projects/BRATS2012) organized by B. Menze, A. Jakab, S. Bauer, M. Reyes, M. Prastawa, and K. Van Leemput. The challenge database contains fully anonymized images from the following institutions: ETH Zurich, University of Bern, University of Debrecen, and University of Utah. All human subjects data was publicly available de-identified data. Therefore, no Institutional Review Board approval was required” [20, 21].

The BRATS database [20] as mentioned above, contains four categories of images:

- High-grade (HG) glioma cases of 20 real subjects,
- Low-grade (LG) glioma cases of 10 real subjects,
- High-grade (SimHG) glioma cases of 25 simulated subjects, and
- Low-grade (SimLG) glioma cases of 25 simulated subjects.

**Fig 2:**
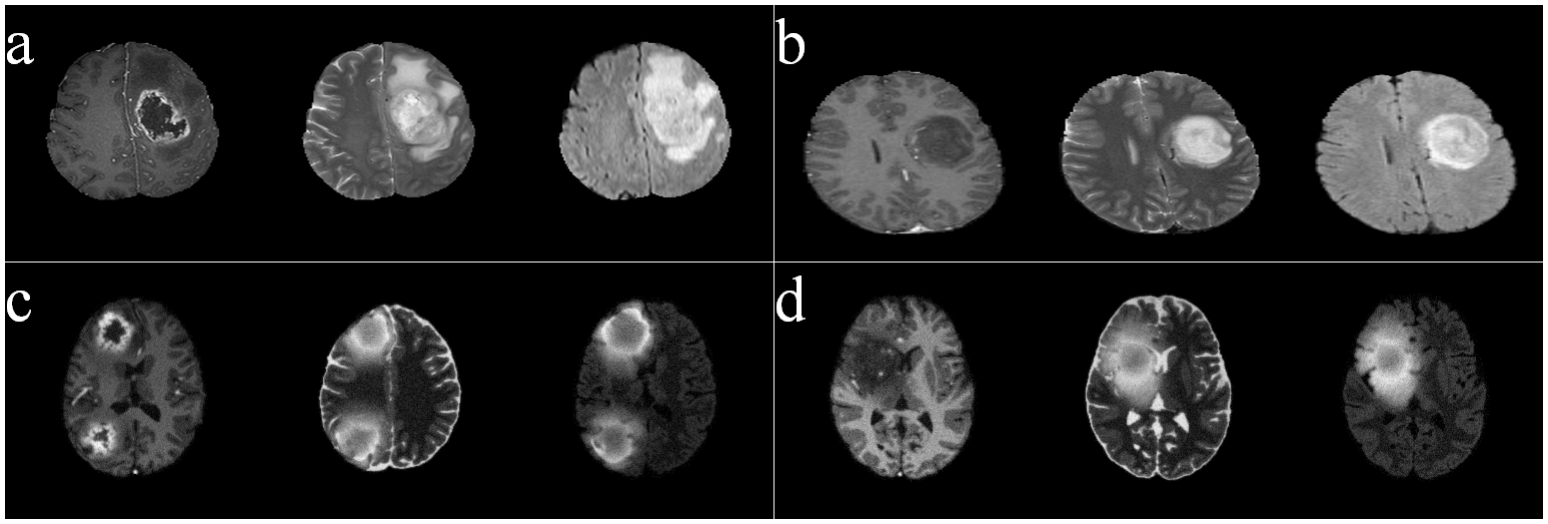
Sample images, of four categories, from BRATS. (a) HG, (b) LG, (c) SimHG, and (d) SimLG.

Fig 2 depicts sample images from these four types, with the three adjacent columns (in each case) corresponding to the sequences *T*1*C*, *T*2, and *FLAIR*, respectively. It is visually obvious that the *T*1*C* sequences of the HG images are hyper-intense in the active tumor region, unlike those of the LG type. All images were linearly co-registered and skull stripped.

We developed a novel saliency detection model for the whole tumor from brain MR images of glioma, using the three channel sequences *viz*. *FLAIR*, *T*2 and *T*1*C* for mapping into a pseudo-color space. The concept of saliency is employed, to enable the algorithm quickly focus on the ROI. Here we use a pseudo-coloring strategy, for MR images, to efficiently generate the saliency map.

### Pseudo-coloring

Digital color images are often constructed from three stacked color channels *viz*. red, green and blue (*RGB*). These can be decomposed to three gray scale images, in six ways, and recomposed back to the *RGB* image. For example, let us consider three gray-scale images *A*, *B*, *C*, and let *A* be assigned to Red, *B* to Green, and *C* to Blue. Then the six combinations are *ABC*, *ACB*, *BAC*, *BCA*, *CAB*, *CBA*.

**Fig 3:**
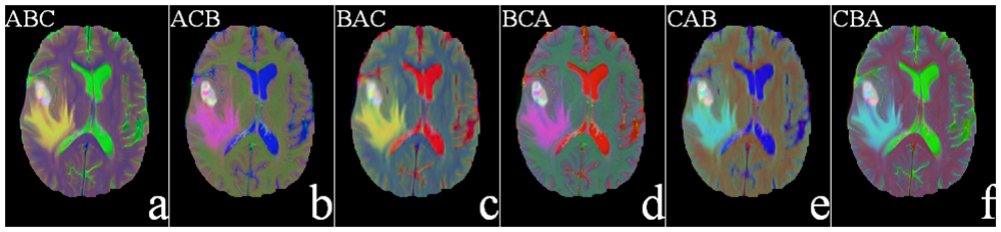
Six pseudo-colored brain MR sequences. (a) *FLAIR*—*T*2—*T*1*C*, (b) *FLAIR*—*T*1*C*—*T*2 (c) *T*2—*FLAIR*—*T*1*C*(d) *T*2—*T*1*C*—*FLAIR*(e) *T*1*C*—*FLAIR*—*T*2, and (f) *T*1*C*—*T*2—*FLAIR*, as mapped to the *RGB* plane.

The proposed pseudo-coloring scheme assigns the three MR sequences (*FLAIR*, *T*2, *T*1*C*) to *RGB* for generating a “color” MR image. These are false colors (or pseudo-colors), and do not correspond to the color of the imaged tissue(s). However, such pseudo-coloring yields a 24 bit MR image containing about 65,536 times more information than a single-channel gray-scale image. Thereby a single color MR image is capable of detecting and displaying the whole tumor region as the ROI from the pseudo-colored images, as displayed in Fig 3.

It is observed from the figure that the tumor appears white in all sequence combinations. This is because it is equally bright in all the three channels of *RGB*. The edema, on the other hand, looks yellow in Fig 3(a) and 3(c) because of its brightness along primary color channels *R*, *G*. Here these correspond to *FLAIR* and *T*2 sequences, and occupy the first two positions in these images.

Since our saliency detection algorithm depends on the center-surround difference of a region with its neighbors, based on the pixel color values, a perceptually uniform color space (distance between any two color is perceived proportional to their distance in the color space) that decorrelates luminance from chrominance information is desirable. Therefore, *RGB* is found to be not that suitable for delineating the tumor regions. In this context the International Commission on Illumination (CIE) recommended the use of *CIE*—L* a* b* for representing color difference, with their first distance metric being Δ*E*_76_. This is the Euclidean distance for quantifying the difference between two color points $V_{i}=L_{i}^{*},a_{i}^{*},b_{i}^{*}$, $V_{j}=L_{j}^{*},a_{j}^{*},b_{j}^{*}$, and formulated as Δ*E*_76_ = ||*V*_*i*_ − *V*_*j*_||, with ||.|| denoting the *L*_2_-norm [22].

**Fig 4:**
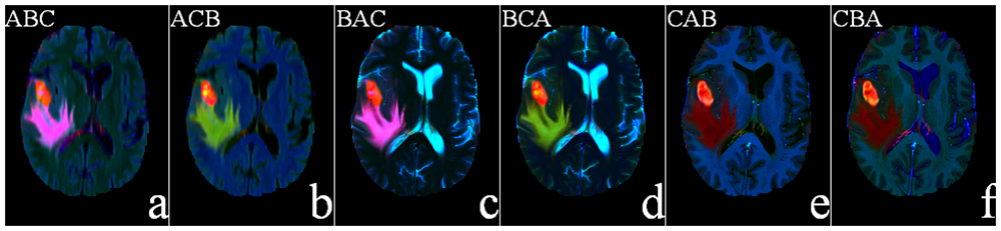
Six pseudo-colored (L* a* b*) converted brain MR image sequences, corresponding to the six pseudo-colored (*RGB*) MR sequences of Fig 3.

We next consider the transformation to the *CIE*—L* a* b* color space for a new way of mapping the brain MR images of glioma. Converting an image into L* a* b* from *RGB*, results in the separation between the two layers, luminosity and chromaticity. The L* a* b* converted MR images, corresponding to Fig 3, are shown in Fig 4.

**Fig 5:**
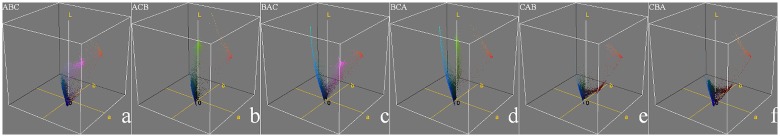
3-D color cubes showing the color distributions of the L* a* b* converted images of Fig 4.

It is visually obvious from the figure that, in the first two sequences, *ABC* and *ACB*(Fig 4(a) and 4(b)), only the tumor and edema regions get highlighted while all other regions are suppressed. The corresponding color cubes are depicted in Fig 5. From Fig 5(a) and 5(b), we find that the principal components representing the tumor (orange) and edema (violet or green) regions are quite large in both cases. This is because *FLAIR* reflects both the tumor and edema regions (albeit, with a fuzzy boundary), and in both Fig 5(a) and 5(b) it occupies the first position in the sequence. Therefore we can use either of these two sequences. In this study we choose the sequence *ABC* for subsequent saliency detection. It may also be noted that *T*1*C* suppresses the edema region in Fig 4(e) and 4(f), while *T*2 also illuminates the CSF in Fig 4(c) and 4(d). This is corroborated from Fig 5(c)–5(f). Hence these four sequences are not considered in our study.

The algorithm for the pseudo-coloring scheme consists of two steps.

**Step 1:** Create a pseudo-colored *RGB* image from the *FLAIR*, *T*2, *T*1*C* MR image.
**Step 2:** Transform from *RGB* color space to *CIE*–L* a* b* color space to enhance local contrast.

This is followed by the generation of local and global contrast-based saliency map for detecting whole tumor regions, as described below.

### Localization of ROI through saliency

In an image a salient region is formed by one (or more) very important piece(s) of composition, to make it stand out from its surroundings. An L* a* b* image (of size *M* × *N*) is first transformed to a square image of size *w* × *w*. Since the database can contain images of different sizes, these need to be converted to one uniform size (preferably a squared one, here *w* = 256). Then it is decomposed into several non-overlapping blocks *R*_*i*_(or patches) of size *k* × *k* pixels (where *w* is a multiple of *k*), with each being represented by its mean L* a* b* values. The number of patches (*w*/*k* × *w*/*k*) correspond to the number of pixels in the saliency map. Let the *i*th patch of the image *I*(*R*_*i*_), 1 ≤ *i*≤ (*w*/*k* × *w*/*k*), be represented by its mean L* a* b* color values as

$$\begin{array}{c} \bar{R_{i}^{L^{*}}}=\frac{\sum I(R_{i}^{L^{*}})}{k\times k},\bar{R_{i}^{a^{*}}}=\frac{\sum I(R_{i}^{a^{*}})}{k\times k},\bar{R_{i}^{b^{*}}}=\frac{\sum I(R_{i}^{b^{*}})}{k\times k}. \end{array}$$ (1)

Next the saliency of each patch is calculated with respect to all other patches in the image. Color is considered as the most important feature in the bottom-up approach, with the simple color difference between regions providing an efficient way to highlight the salient region(s) with respect to the non-salient patches. The color difference between a pair of patches is defined as the “Euclidean distance” between the corresponding mean color values of L* a* b*. Therefore, for patch *R*_*i*_, the saliency *S*_*c*_(*R*_*i*_) is calculated as the sum of the color difference between $\bar{R_{i}^{L^{*}}}$, $\bar{R_{i}^{a^{*}}}$, $\bar{R_{i}^{b^{*}}}$ and $\bar{R_{j}^{L^{*}}}$, $\bar{R_{j}^{a^{*}}}$, $\bar{R_{j}^{b^{*}}}$(Eq (1)), ∀ *j* ≠ *i*. It is expressed as

$$\begin{array}{c} S_{c}\left(R_{i}\right)=\sum_{j,j\neq i}\sqrt{{\left(\bar{R_{i}^{L^{*}}}-\bar{R_{j}^{L^{*}}}\right)}^{2}+{\left(\bar{R_{i}^{a^{*}}}-\bar{R_{j}^{a^{*}}}\right)}^{2}+{\left(\bar{R_{i}^{b^{*}}}-\bar{R_{j}^{b^{*}}}\right)}^{2}}\\ \forall i,j\in \{1,\ldots ,(w/k\times w/k)\}. \end{array}$$ (2)

The color difference of a patch with the rest of the patches in the image is summed. If this sum is large then it is considered to be a salient patch. Typically while most salient patches are observed to be concentrated around spatially adjacent areas, the other (non-salient) patches may be distributed anywhere over the whole image. If a region is salient then the probability is large for its surrounding regions to be salient, while the probability of those regions located farther away from it being salient becomes small. Therefore the influence of adjacent regions can be considered to be more important when computing the saliency of a region. Keeping this in view we incorporate the spatial distance between patches as another important factor for calculating image saliency. In the process, we consider (i) the difference of the L* a* b* color values between any two blocks, and (ii) the spatial distance between them. Now Eq (2) gets redefined as

$$\begin{array}{ccc} S(R_{i}) & = & \sum_{j,j\neq i}\frac{1}{1+d(R_{i},R_{j})}\times S_{c}\left(R_{i}\right). \end{array}$$ (3)

Here $d\left(R_{i},R_{j}\right)=\sqrt{{\left({\bar{x}}_{R_{i}}-{\bar{x}}_{R_{j}}\right)}^{2}+{\left({\bar{y}}_{R_{i}}-{\bar{y}}_{R_{j}}\right)}^{2}},$ where *d*(*R*_*i*_, *R*_*j*_) is the spatial distance between the patches *R*_*i*_ and *R*_*j*_ of the image, and (${\bar{x}}_{R_{i}}$, ${\bar{y}}_{R_{i}}$) refers to the mean spatial coordinates of *R*_*i*_.

**Fig 6:**
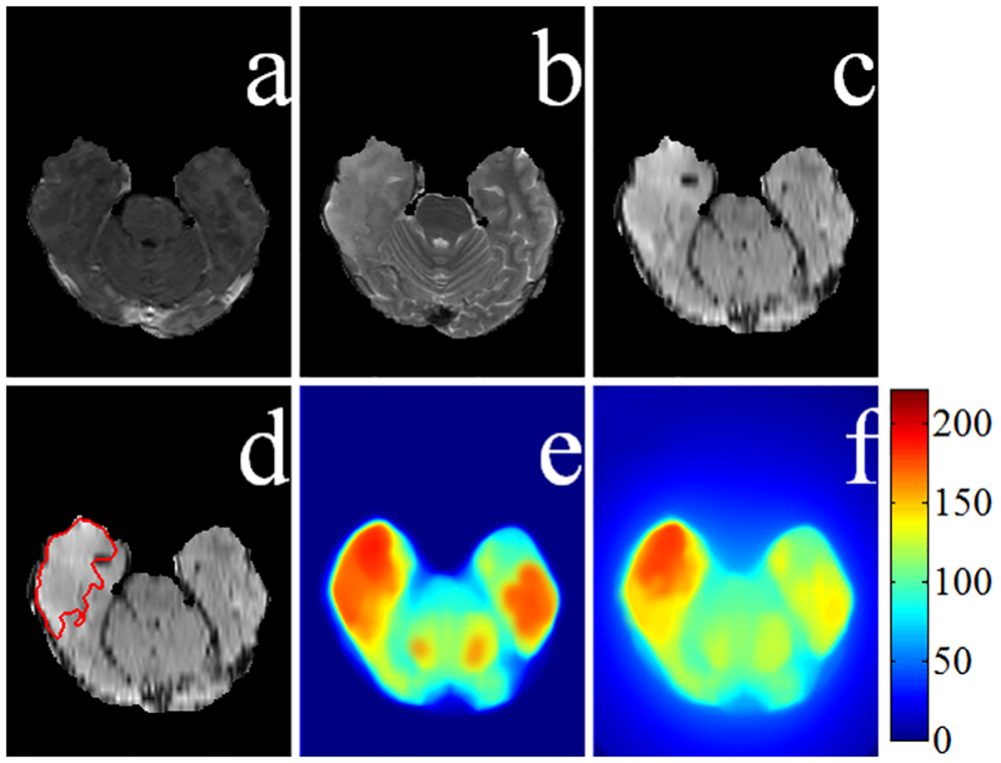
Saliency map generation for a low-grade glioma. MRI sequences (a) *T*1*C*, (b) *T*2, and (c) *FLAIR*. (d) Ground truth superimposed on the *FLAIR* image. Saliency maps (e) without spatial distance, and (f) with spatial distance component.

Let us illustrate the situation with MR sequences of a low-grade glioma in Fig 6. While Fig 6(a)–6(c) depict the *T*1*C*, *T*2, and *FLAIR* sequences, respectively, Fig 6(d) indicates the ground truth about the tumor (including active tumor and edema regions) in the *FLAIR* mode. Fig 6(f) demonstrates a more accurate (lower false positive) detection of the salient region in the pseudo-colored space, after incorporating the spatial distance component by Eq (3), as compared to Fig 6(e). Since the contrast between the tumor and normal tissue regions is not very large in low-grade glioma images, often the sum of color difference of a patch with respect to all other patches in the image remains large at several locations. This results in an incorrect detection of multiple salient regions by Eq (2).

Now consider an observer viewing a far-off scene. The focus lies on the entire salient region(s). Again when the same scene is viewed at a closer range, the observer tends to pay more attention to the details within the salient region [6, 23]. We adopted this property of the human visual attention mechanism into our model through the evaluation of multiple-scale based saliency maps. By partitioning an image into smaller sized patches, we can clearly highlight the salient object along with its details.

**Fig 7:**
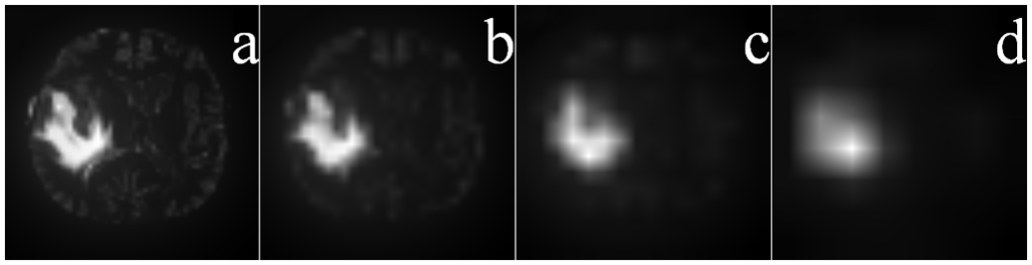
Saliency map of pseudo-colored MR image of the whole tumor region of Fig 4(a) at different scales. Patch sizes of (a) 4 × 4, (b) 8 × 8, (c) 16 × 16, and (d) 32 × 32.

Although the saliency map for a larger patch can help in accurately locating a salient object, its resultant blurring causes disappearance of most details. Saliency maps, depicting the saliency strength at every pixel over different scales, involving varying sizes of the patches (with *k* = 4, 8, 16, 32, of sizes 4 × 4, 8 × 8, 16 × 16 and 32 × 32) are provided in Fig 7 (after rescaling these to their original sizes). These images correspond to the tumor and edema regions of Fig 4(a). Comparing Fig 7(a)–7(d) we observe that as the contour of the ROI gets gradually blurred, with increasing patch size *k*, the position of the salient region becomes clearer. Here the block size *k* relates to the resolution of the saliency map.

**Fig 8:**
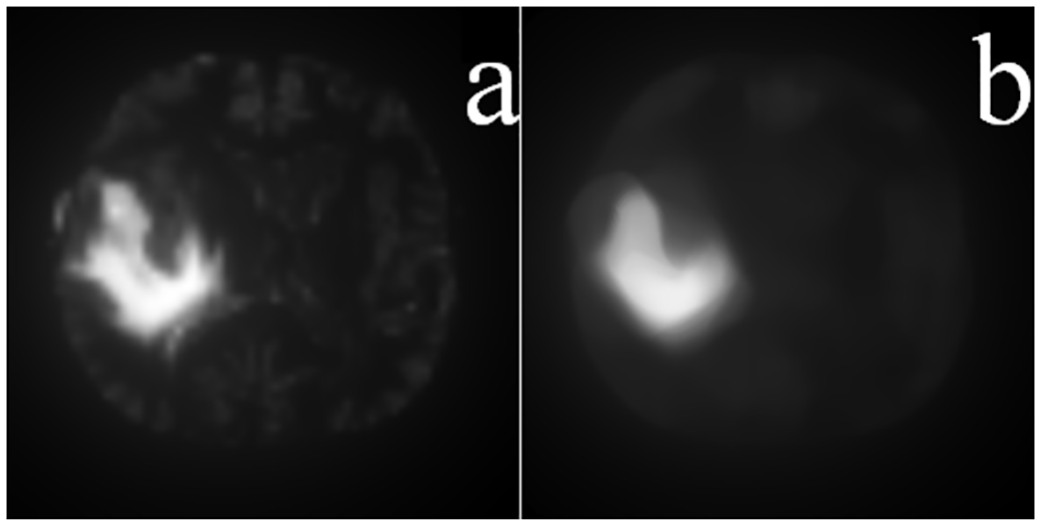
Final saliency map. Superimposed saliency map corresponding to Fig 7 and (b) its final smoothened version.

Next, a re-scaling is performed to bring back the saliency maps to the original image size (*M* × *N*) using Bilinear interpolation [23]. Let ${\hat{S}}^{k}$ denote the interpolated image at its original size, as generated from the saliency map *S*^*k*^ at scale *k*. Since the properties of a region depend on the pixels within it, saliency prediction is related to size and scale of the region on which detection is performed. Our algorithm is employed simultaneously over multiple scales, analogous to [23], for capturing the salient region(s) in the *MR* image at different levels of resolution. Those region(s) consistently highlighted over different resolutions are assumed to be the ones most likely to be salient. Therefore we superimpose these saliency maps, corresponding to the different scales, for computing the final map. For example, the integrated map over the four scales (of Fig 7) contains all important information and is depicted in Fig 8(a). The final saliency map is now computed as

$$\begin{array}{c} S=\sum_{k=4,8,16,32}r^{k}\times {\hat{S}}^{k}, \end{array}$$ (4)

where *r*^*k*^ is the weight corresponding to the saliency map at size *k*. In the present study we have chosen *r*^*k*^ = 1/4, ∀ *k*. Finally a 25 × 25 mean filter is applied to smoothen the saliency map *S*, in order to help focus on the core region within the actual ROI in the resized image. This is depicted in Fig 8(b), and acts as the reference map for subsequent segmentation.

In summary, our contribution lies in introducing the L* a* b* pseudo-color space along with the spatial distance, while computing the saliency at multiple scales.

The algorithm combines extracts saliency maps in multiple scale to generate one final saliency map using a fusion strategy. The underlying assumption about spatial coincidence identifies a region as salient only if it is found to be consistently salient over multiple scales.

## Experimental Results and Discussion

The performance of the proposed saliency detection model (PR), as well as those of four state-of-the-art algorithms from literature (Itti [6], SIM [12], COV [23], and SDSP [24]), are evaluated in terms of the saliency map by comparing it with the ground truth of the whole tumor region encompassing the intra-tumoral structures, namely “edema”, “nonenhancing (solid) core”, “necrotic (or fluid-filled) core”, and “non-enhancing core” as demarcated by expert radiologists and using several performance metrics. The performance of the algorithms was evaluated both qualitatively and quantitatively.

A saliency map is represented as an gray scale image of the same size as that of original image, with the intensity of a pixel indicating its importance for belonging to the tumor region in the original image. While an intensity 0 (pure black) indicates least importance, an intensity of 255 (pure white) corresponds to highest importance. We first generate binary masks for the salient object by thresholding the saliency map, over varying thresholds ranging from 0 to 255. These are compared with the ground truth, based on different metrics.

Precision refers to the percentage of correctly classified salient pixels over the whole image, whereas recall corresponds to the portion of pixels from the ground truth which get detected correctly. Although recall and precision vary at the cost of each other, both the measures are important. Therefore we have maximized both of these. The entire range of gray levels in the image is explored, for exhaustive thresholding, in order to generate two classes; with the positive class representing the ROI and the negative class being treated as the background. Area under the curve (AUC) is estimated by analyzing the receiver operator characteristic (ROC) from these thresholded images. While the true positive rate (TPR) is the proportion of saliency values at actual location above a threshold, the proportion of pixels corresponding to the non-tumorous regions of the ground truth (but wrongly classified as tumor regions) contribute towards the false positive rate (FPR).

The performance of our algorithm is evaluated by computing the precision and recall, along with the TPR and FPR over these thresholded maps. The precision-recall and ROC curves are plotted, by averaging over the set of images for each data group (HG LG, SimHG, and SimLG). The corresponding mean AUC scores (with standard deviation) and *p*-values using independent samples t-test while comparing our algorithm with four other state-of-the-art methods, are presented in Table 1.

**Table 1:** Comparison of mean AUC scores with considered saliency models.

Data group	AUC score (Mean ± SD)				
	Itti [6]	SIM [12]	SDSP [24]	COV [23]	PR
HG	0.824 ± 0.121 (p <0.01)	0.921 ± 0.041 (p <0.01)	0.956 ± 0.032 (p <0.01)	0.891 ± 0.069 (p <0.01)	**0.996 ± 0.002**
LG	0.906 ± 0.080 (p <0.01)	0.913 ± 0.066 (p <0.01)	0.976 ± 0.019 (p <0.01)	0.929 ± 0.032 (p <0.01)	**0.998 ± 0.001**
SimHG	0.916 ± 0.060 (p <0.01)	0.932 ± 0.042 (p <0.01)	0.963 ± 0.012 (p <0.01)	0.974 ± 0.014 (p <0.01)	**0.998 ± 0.003**
SimLG	0.892 ± 0.072 (p <0.01)	0.925 ± 0.041 (p <0.01)	0.938 ± 0.031 (p <0.01)	0.966 ± 0.018 (p <0.01)	**0.999 ± 0.001**

**Fig 9:**
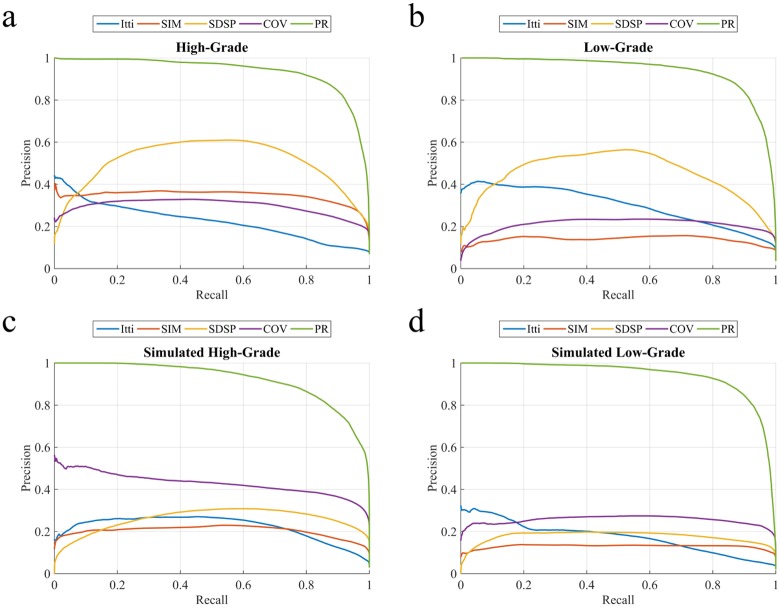
Comparative study of averaged precision-recall values, with varying thresholds [0—255] on saliency map for four groups of MR images. (a) HG, (b) LG, (c) SimHG, and (d) SimLG.

**Fig 10:**
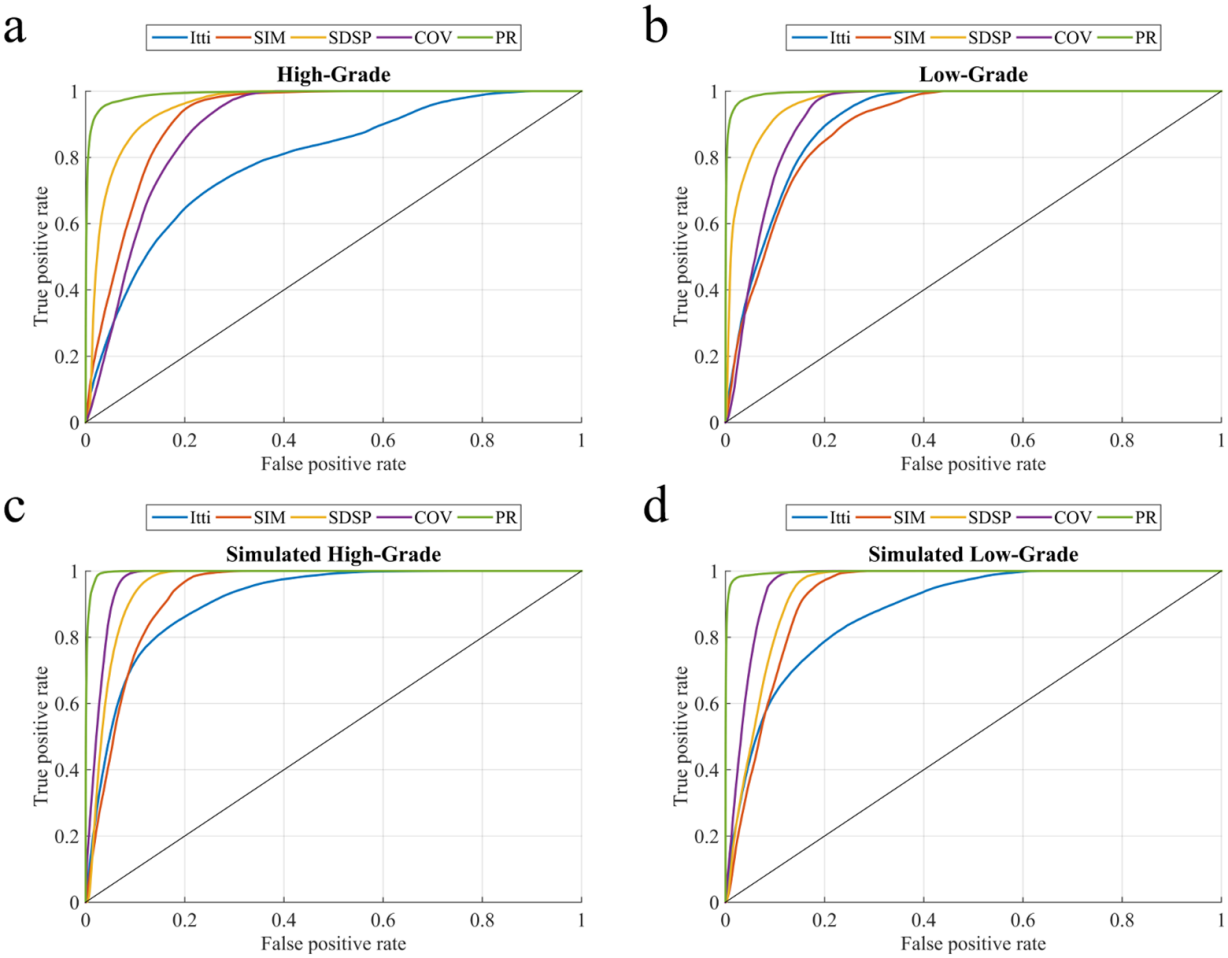
Comparative study of ROC curves for averaged TPR-FPR values, with varying thresholds [0—255] on saliency map for four groups of MR image. (a) HG, (b) LG, (c) SimHG, and (d) SimLG.

The four saliency detection methods compared here, viz. Itti [6], SIM [12], COV [23], and SDSP [24], were originally developed for detecting saliency in natural images. Here we apply these on the pseudo-colored RGB MR images. Ref. [6] generates saliency map based on feature integration, with the feature maps at lower level being created by decomposing the visual inputs. The final saliency map is generated by combining all lower level feature maps, using a weighting scheme. “Saliency Estimation Using a Non-Parametric Low-Level Vision Model” (SIM) uses Wavelet transform, in visual attention modelling, to outperform considered models [12]. “Visual saliency estimation by nonlinearly integrating features using region covariances” (COV) compares covariances of non-overlapping neighbouring image regions, using meta-features to estimate the contribution of different feature dimensions towards the overall visual saliency [23]. “A Novel Saliency Detection Method by Combining Simple Priors” (SDSP) [24] detects the salient region in an image by integrating three simple priors, viz. frequency, location and L*a*b* color space to generate the final saliency map. The experimental results are illustrated for each of the four categories of MR images. Fig 9 depicts the precision-recall plots, while Fig 10 shows the corresponding ROC curves. It is observed that the PRoposed model (PR) clearly outperforms the other algorithms. Table 1 presents the comparative AUC scores for each case, with PR again providing the best results (*p*-value < 0.01, with best scores highlighted).

**Fig 11:**
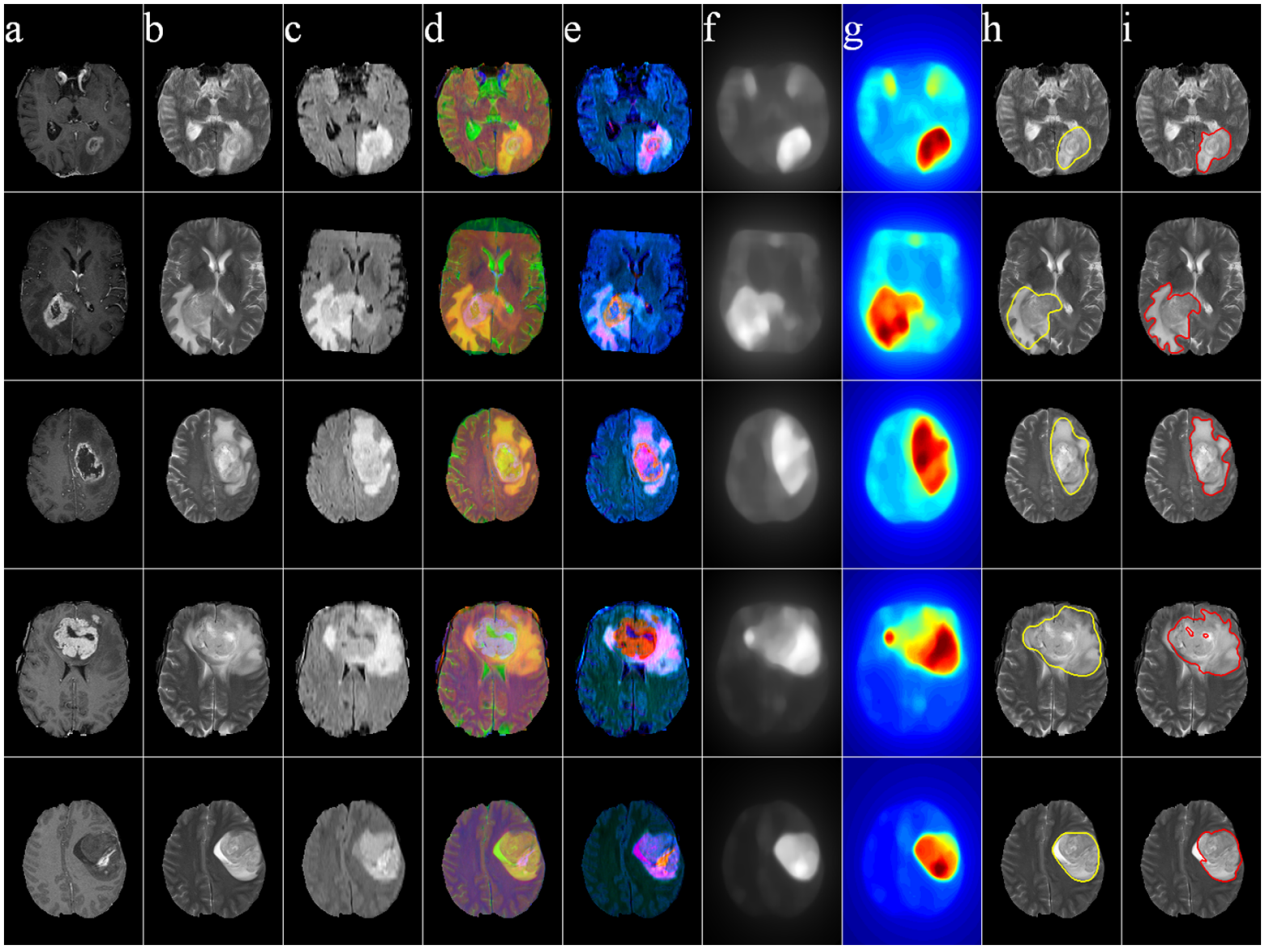
MR images of five High-Grade Glioma cases. (a) T1C, (b) T2, (c) FLAIR, (d) pseudo RGB, and (e) L* a* b*, (f) Saliency map, (g) color coded saliency map, with extracted (h) proto-object, and (i) corresponding ground truth.

**Fig 12:**
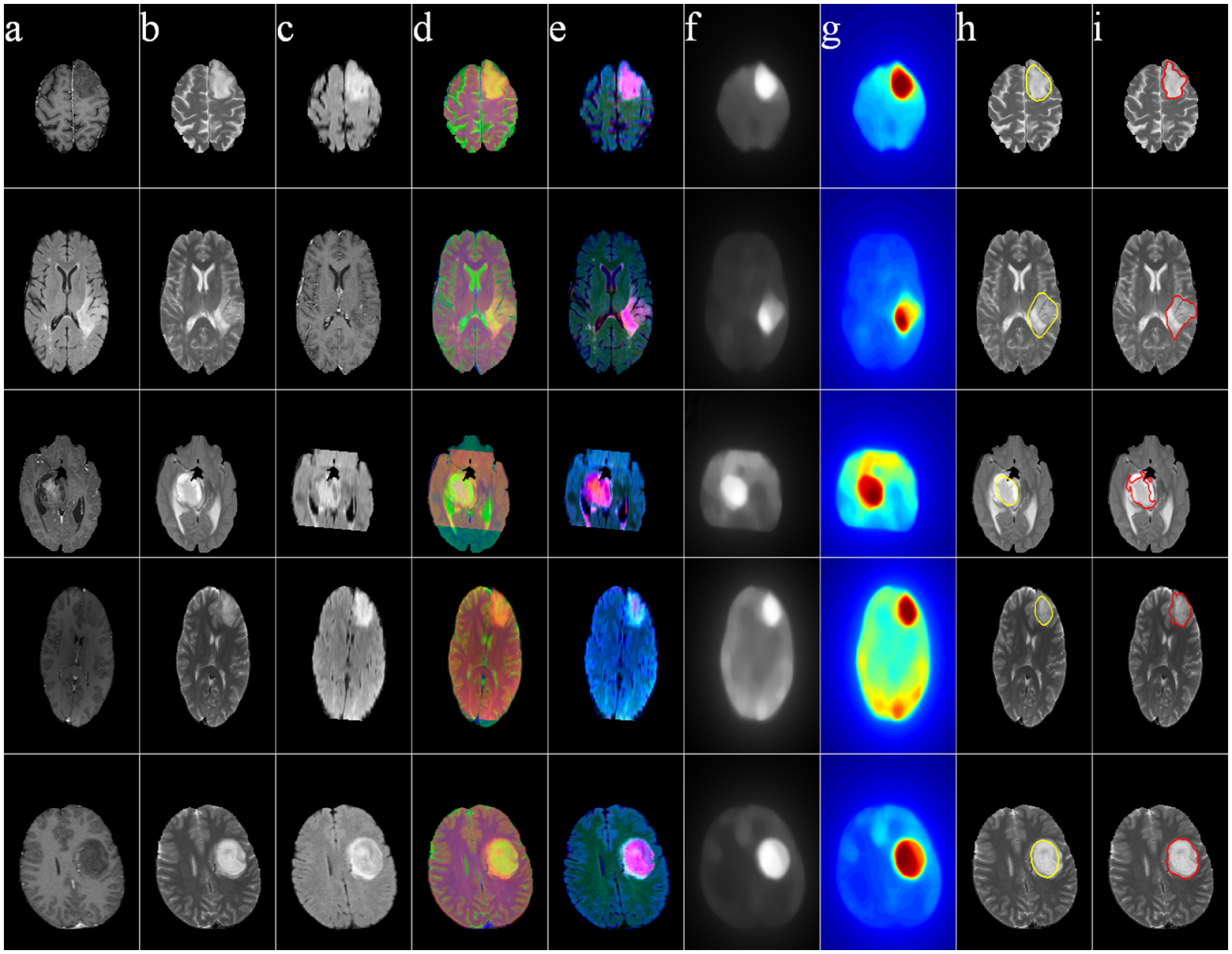
MR images of five Low-Grade Glioma cases. (a) T1C, (b) T2, (c) FLAIR, (d) pseudo RGB, and (e) L* a* b*, (f) Saliency map, (g) color coded saliency map, with extracted (h) proto-object, and (i) corresponding ground truth.

**Fig 13:**
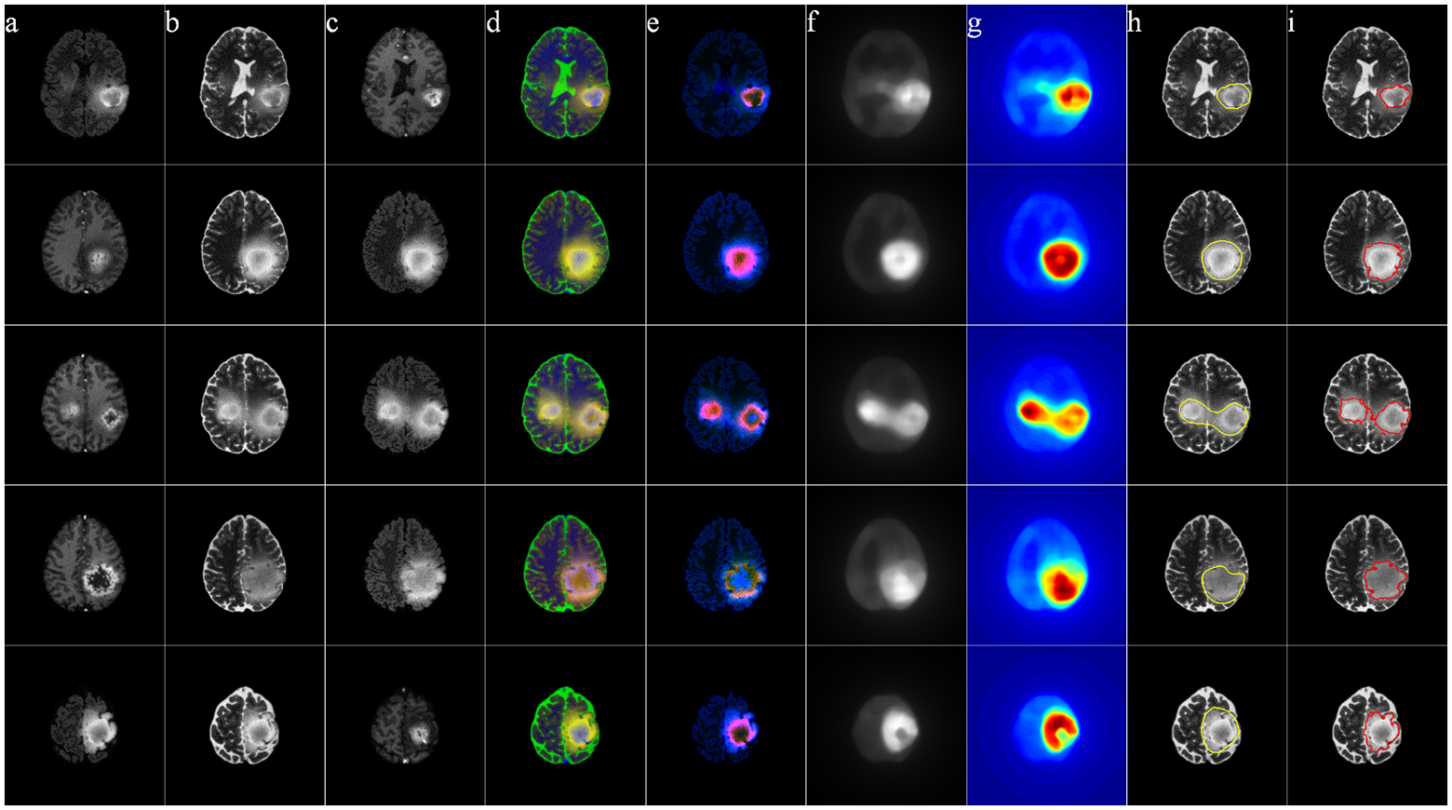
MR images of five Simulated High-Grade Glioma cases. (a) T1C, (b) T2, (c) FLAIR, (d) pseudo RGB, and (e) L* a* b*, (f) Saliency map, (g) color coded saliency map, with extracted (h) proto-object, and (i) corresponding ground truth.

**Fig 14:**
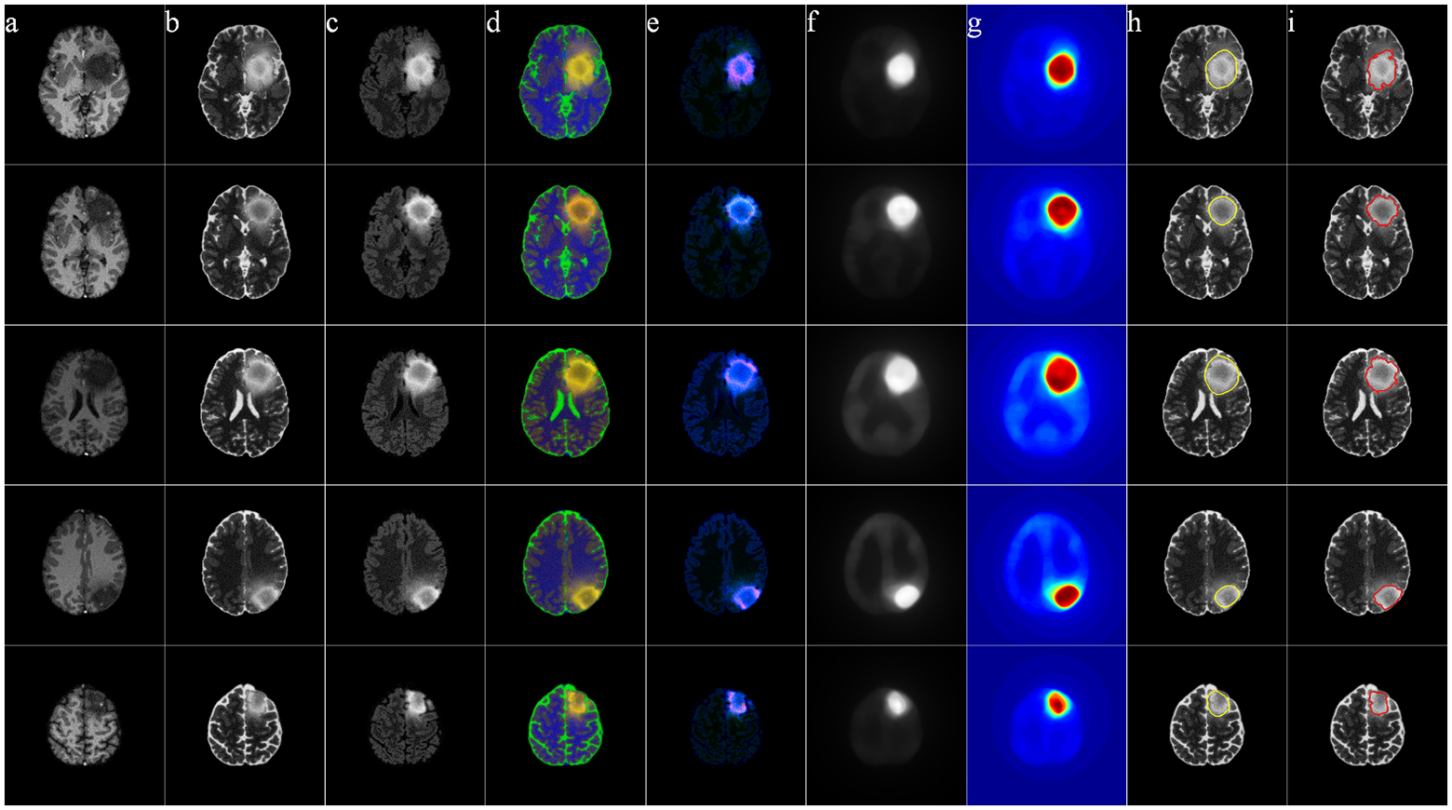
MR images of five Simulated Low-Grade Glioma cases. (a) T1C, (b) T2, (c) FLAIR, (d) pseudo RGB, and (e) L* a* b*, (f) Saliency map, (g) color coded saliency map, with extracted (h) proto-object, and (i) corresponding ground truth.

Figs 11–14 illustrate The visual results on 25 patients, five from each of the four groups HG, LG, SimHG, SimLG. The generat saliency maps *S* by Eq (8) are adaptively thresholded by *T*_*a*_ to generate binary proto-objects which act as prototypes for subsequent segmentation (by any suitable algorithm like region growing or active contour). The threshold is computed as

$$\begin{array}{c} T_{a}=\frac{\alpha }{M\times N}\sum_{x=0}^{M-1}\sum_{y=0}^{N-1}S\left(x,y\right), \end{array}$$ (5)

where *S*(*x*, *y*) denotes the saliency value at location (*x*, *y*) of the image, and *α* is an user-defined parameter empirically set at 2 for high-grade and 3 for low-grade GBM. The qualitative analysis of the extracted ROI establishes that our method is robust to tumor size, shape, position, as well as scanner type.

We observed, there exists no fully automated algorithm to identify the ROI without any prior training or supervision (to best of our knowledge). Typically a supervised identification of the ROI is followed by the application of appropriate operations like enhancement, feature extraction and /or segmentation. Our algorithm, on the other hand, is able to quickly focus on the ROI (in an automated and unsupervised manner) based on the principle of saliency. This can be followed by the application of any suitable segmentation algorithm to extract the ROI(s). The computational cost of our algorithm is very low, i.e.,

$$T=\sum_{k=4,8,16,32}{\left(M/k\right)}^{2}+C≃O\left(M^{2}\right),$$ (6)

from Eq (6), with *C* a constant. Therefore it can be easily implemented in real time or inter-operative environment.

We used *MATLAB* 2014, on an *i*7 CPU having 3.40*GHz* clock speed and 16*GB* of RAM, for our implementation. This was followed by segmentation over the detected ROI of a 2*D* slice. A 3*D* saliency map and its corresponding segmentation can be obtained by repeating the above process over each pseudo colored 2*D* MR slice.

The yellow contour in column (h) in each of Figs 11–14 is generated by thresholding the saliency maps by *T*_*a*_. This can be used as the initial contour in case of active contour based segmentation, or as the seed in case of region growing based techniques. We have not included any quantitative measures to comparatively evaluate the segmentation accuracy of our results, as our contribution lies in the use of saliency for accurately locating the prototype ROI for subsequent segmentation by any existing technique.

## Conclusions

We have designed a novel scheme for saliency detection towards the delineation of whole tumor regions from multi-channel brain MR images. The concept of pseudo-coloring helped in suppressing the less relevant regions, while enhancing its salient parts. The resulting saliency map was compared with four state-of-the-art saliency detection models with respect to the ground truth. Our algorithm provided superior performance, as compared to the considered models, as evident from the experimental results. The AUC score in the ROC analysis was found to range between 0.997 for SimLG and 0.992 for HG images, on an average; which is very high as compared to the existing models.

It may be noted [20] that the identification and segmentation of tumor core region(s) in high-grade glioma is comparatively easier due to contrast enhancement in the *T*1*C* MR sequence. For low-grade glioma, on the other hand, identification of tumor core becomes more challenging and difficult due to the absence of enhancement in its *T*1*C* sequence. Given that our method produces significantly higher detection scores over related methods in case of low-grade glioma, it holds promise towards better diagnosis and/or prognosis of patients having the disease.

## Supporting Information

S1 Fig Additional visual examples of saliency maps by four state-of-the-art models and our proposed model. (PDF)
