# Supplementary material for: A Novel GBM Saliency Detection Model Using Multi-Channel MRI
Source: PLoS One. 2016 Jan 11;11(1):e0146388. doi: 10.1371/journal.pone.0146388 (PMC4709039; doi:10.1371/journal.pone.0146388)

Supporting Information

A Novel GBM Saliency Detection Model using Multi-channel MRI

Subhashis Banerjee, Sushmita Mitra, B. Uma Shankar, Yoichi Hayashi

S1 Fig. Additional visual examples of saliency maps by four state-of-the-art models and our proposed model

Saliency maps for 2D MR slices on HG GBM

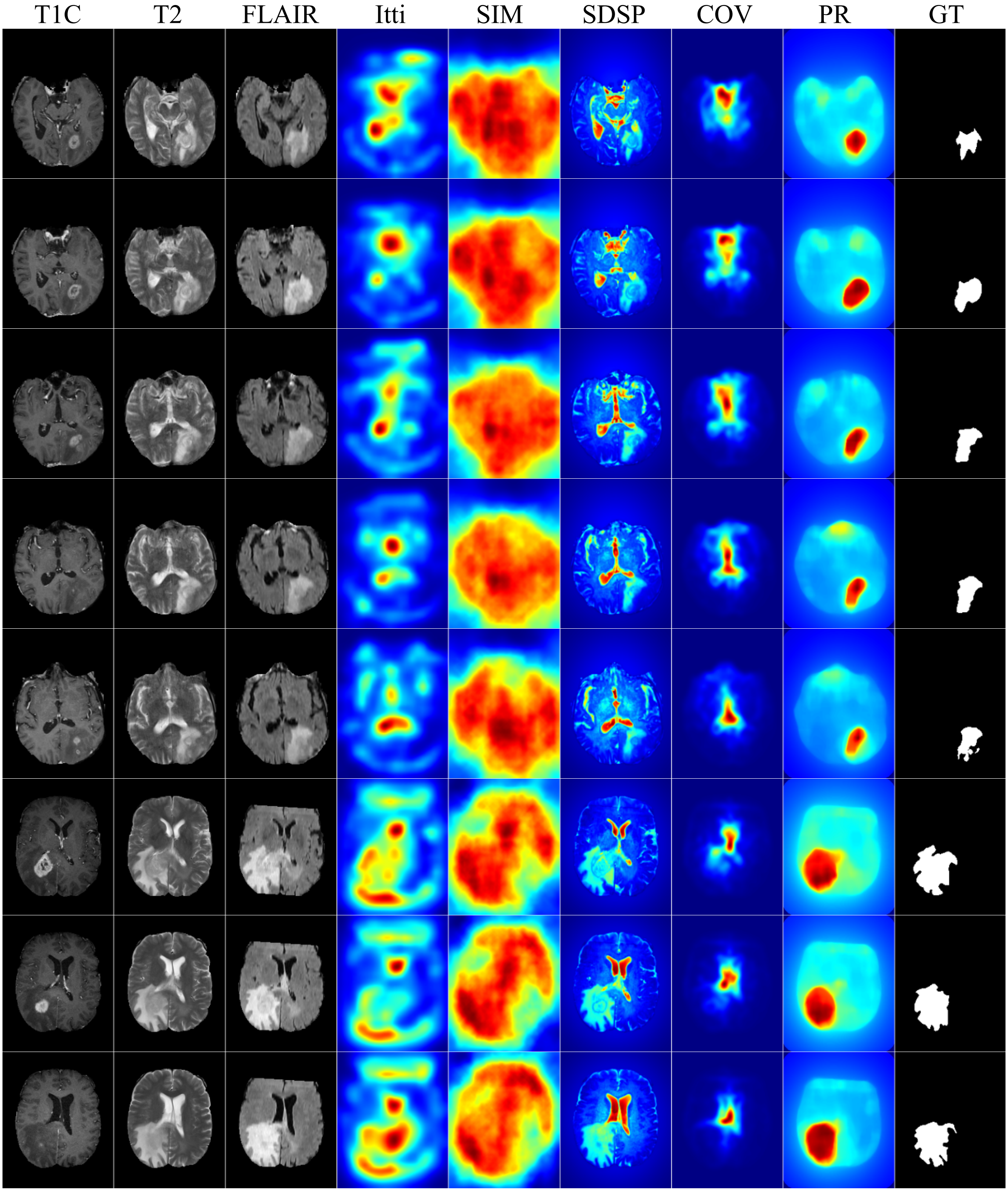

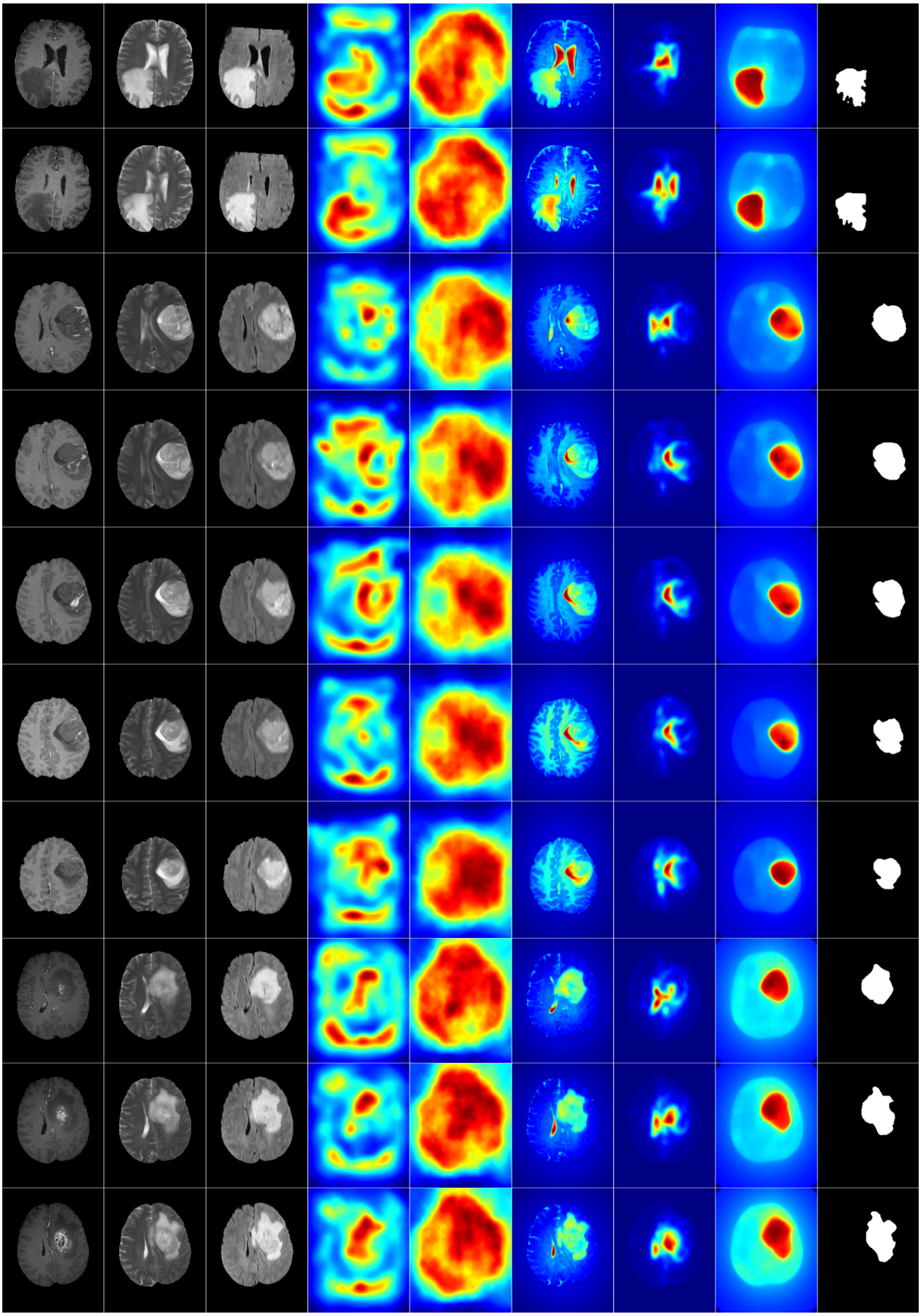

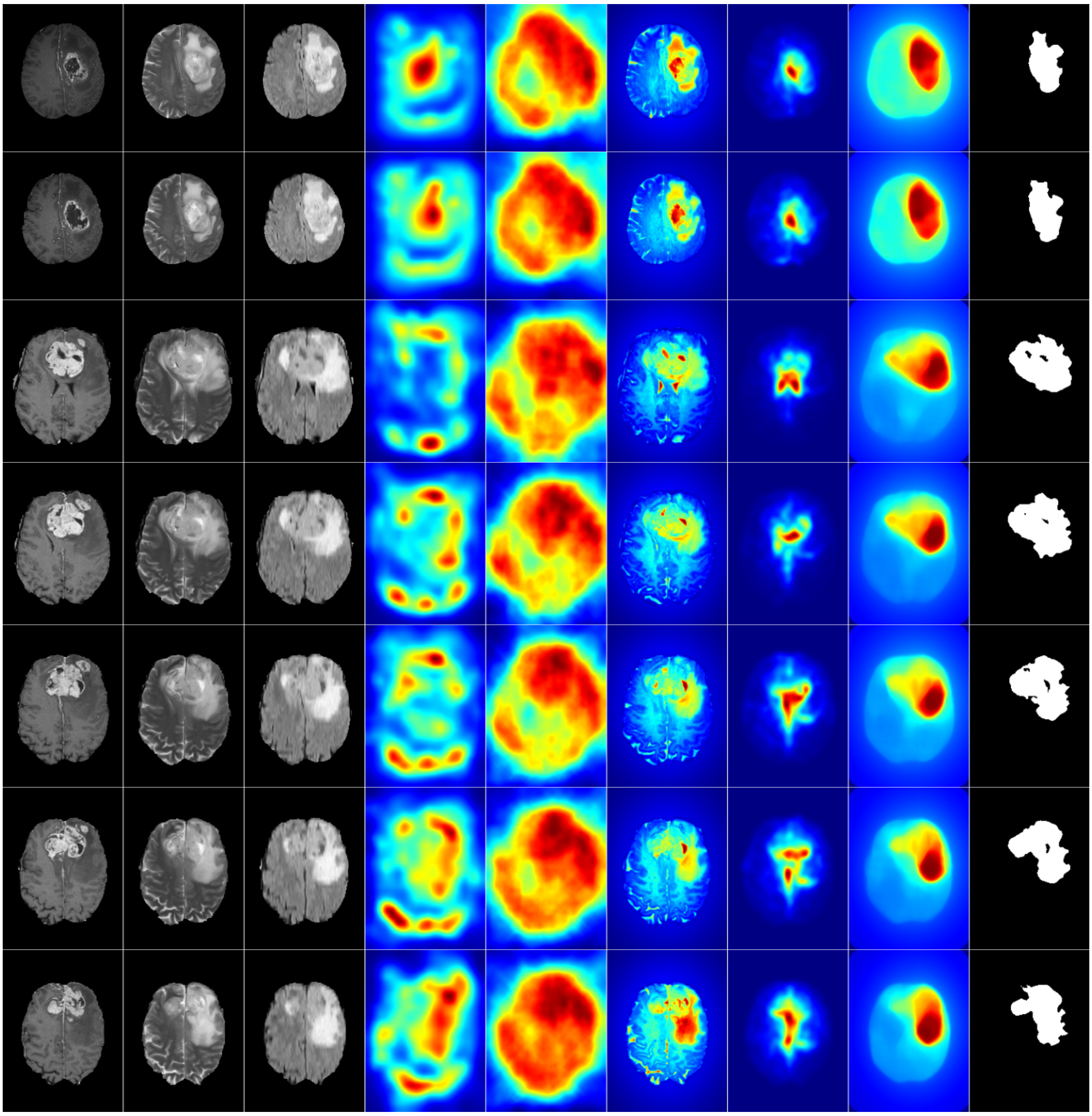

Saliency maps for 2D MR slices with LG GBM

T1C T2 FLAIR Itti SIM SDSP COV PR GT

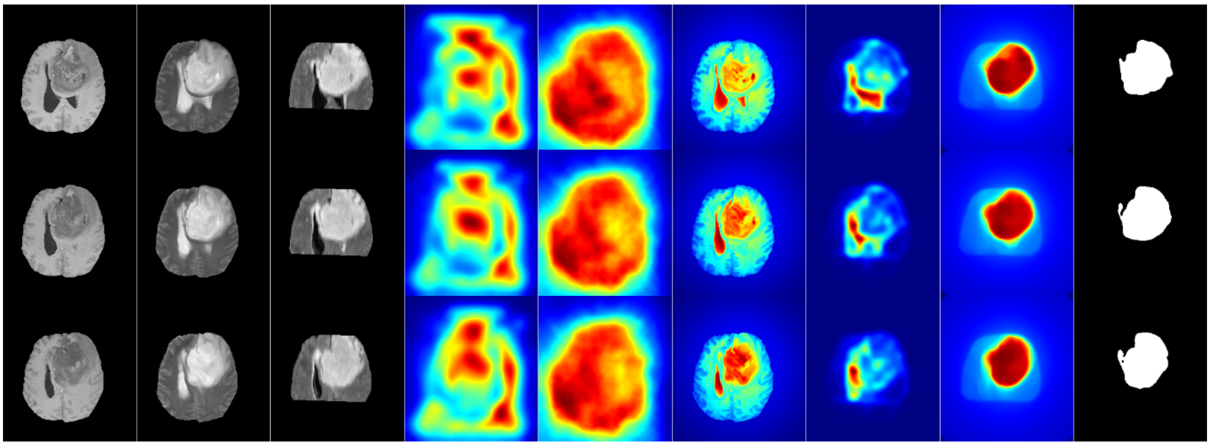

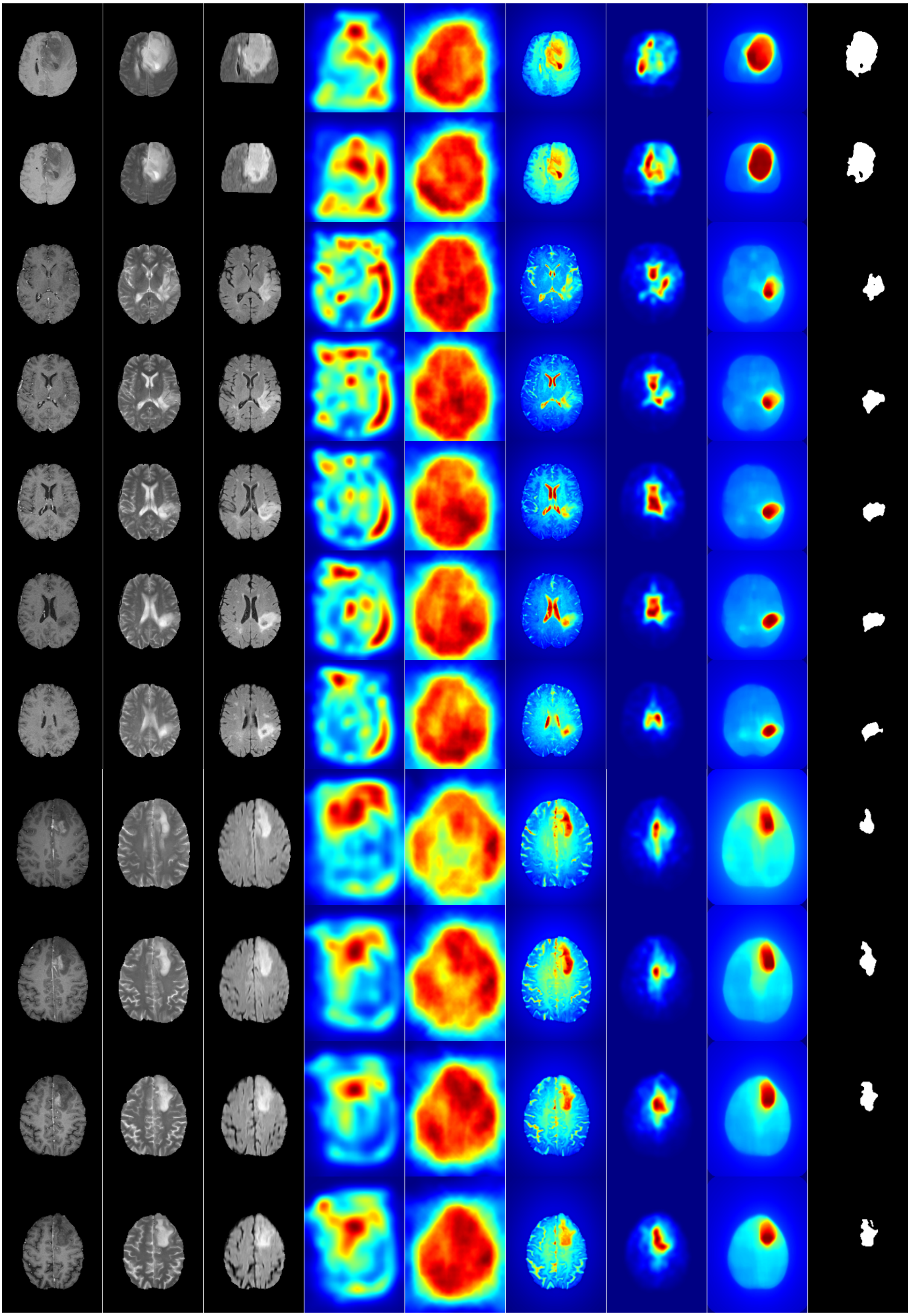

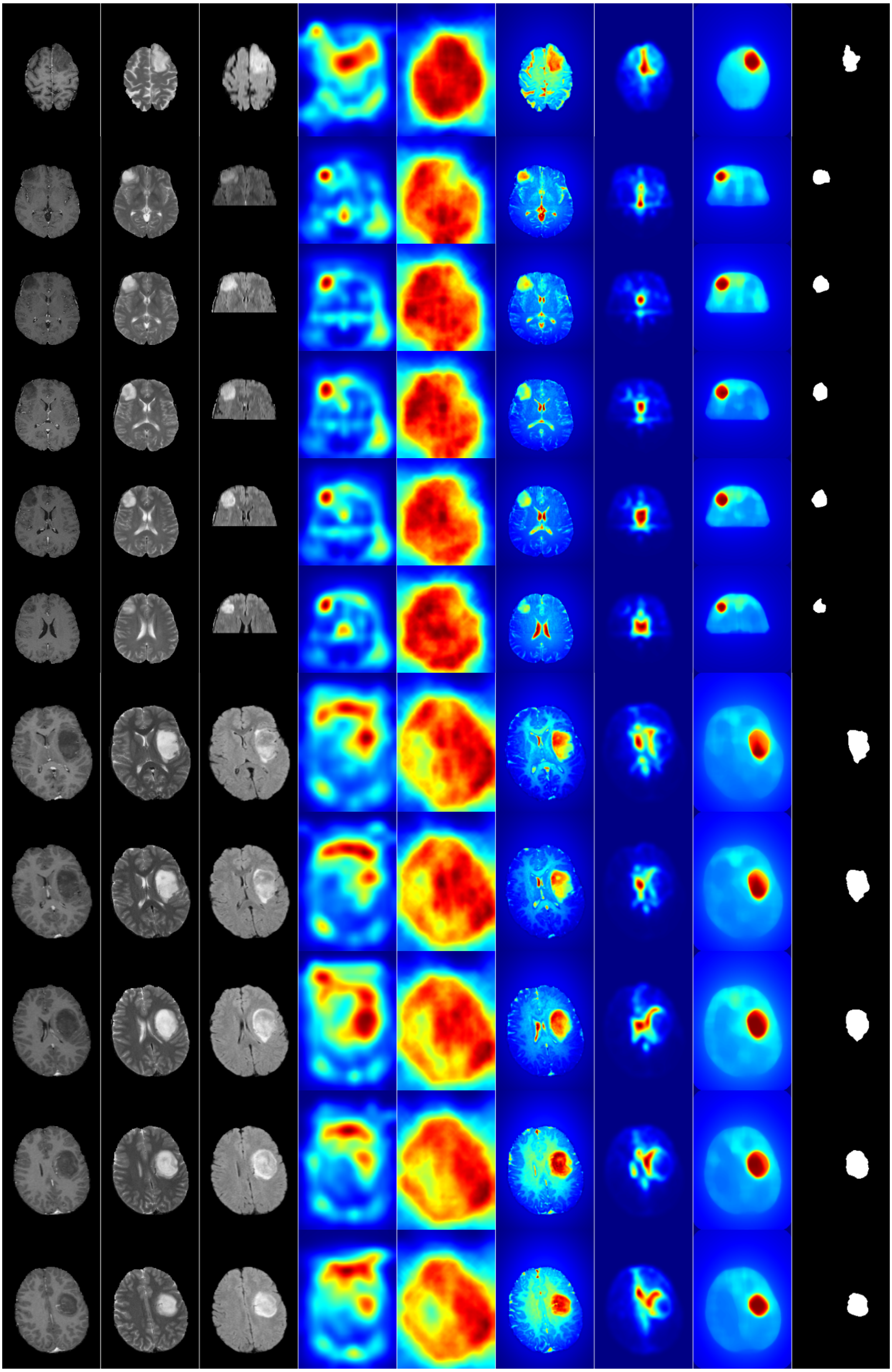

Saliency maps for 2D MR slices with SimHG GBM

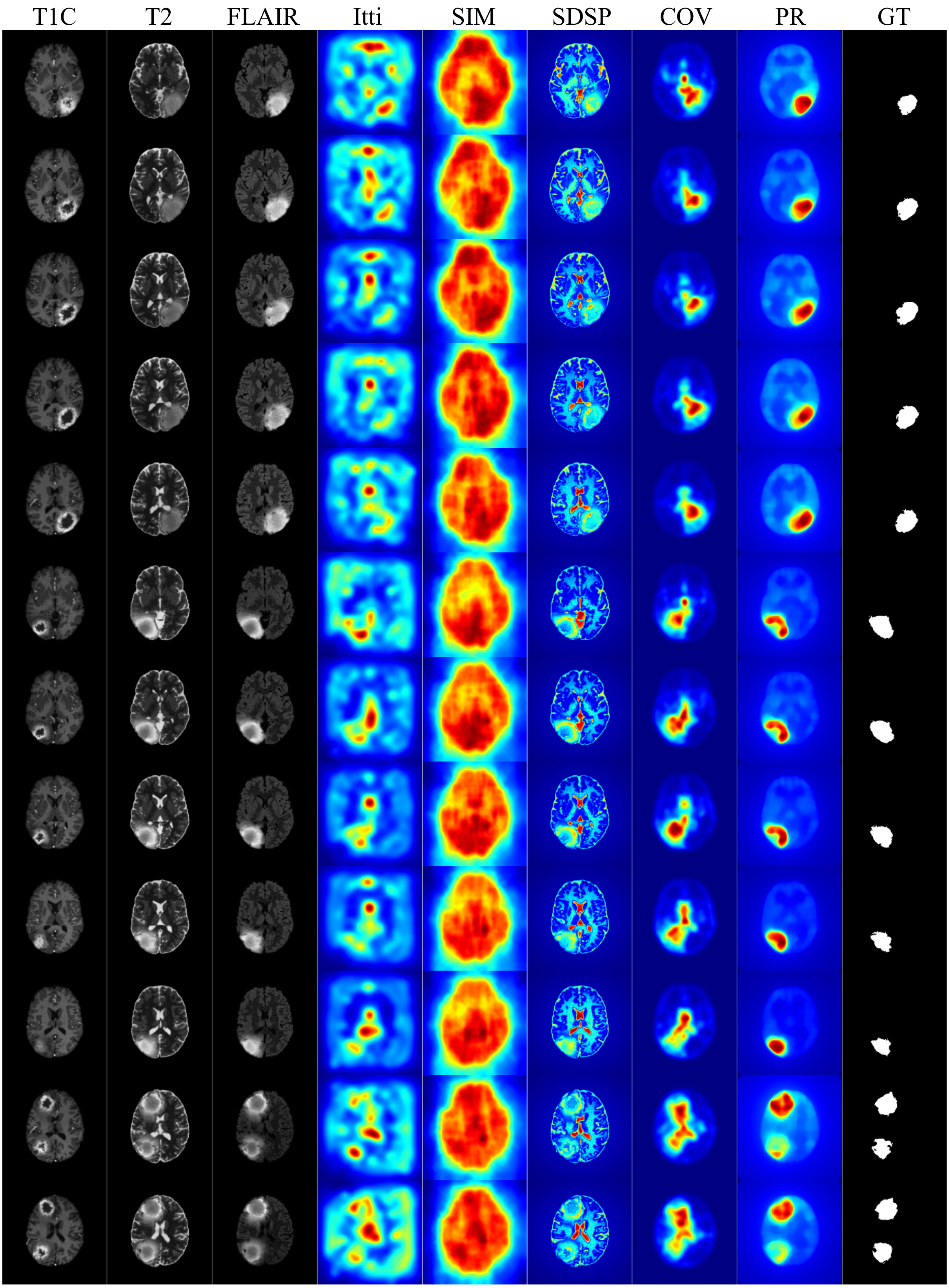

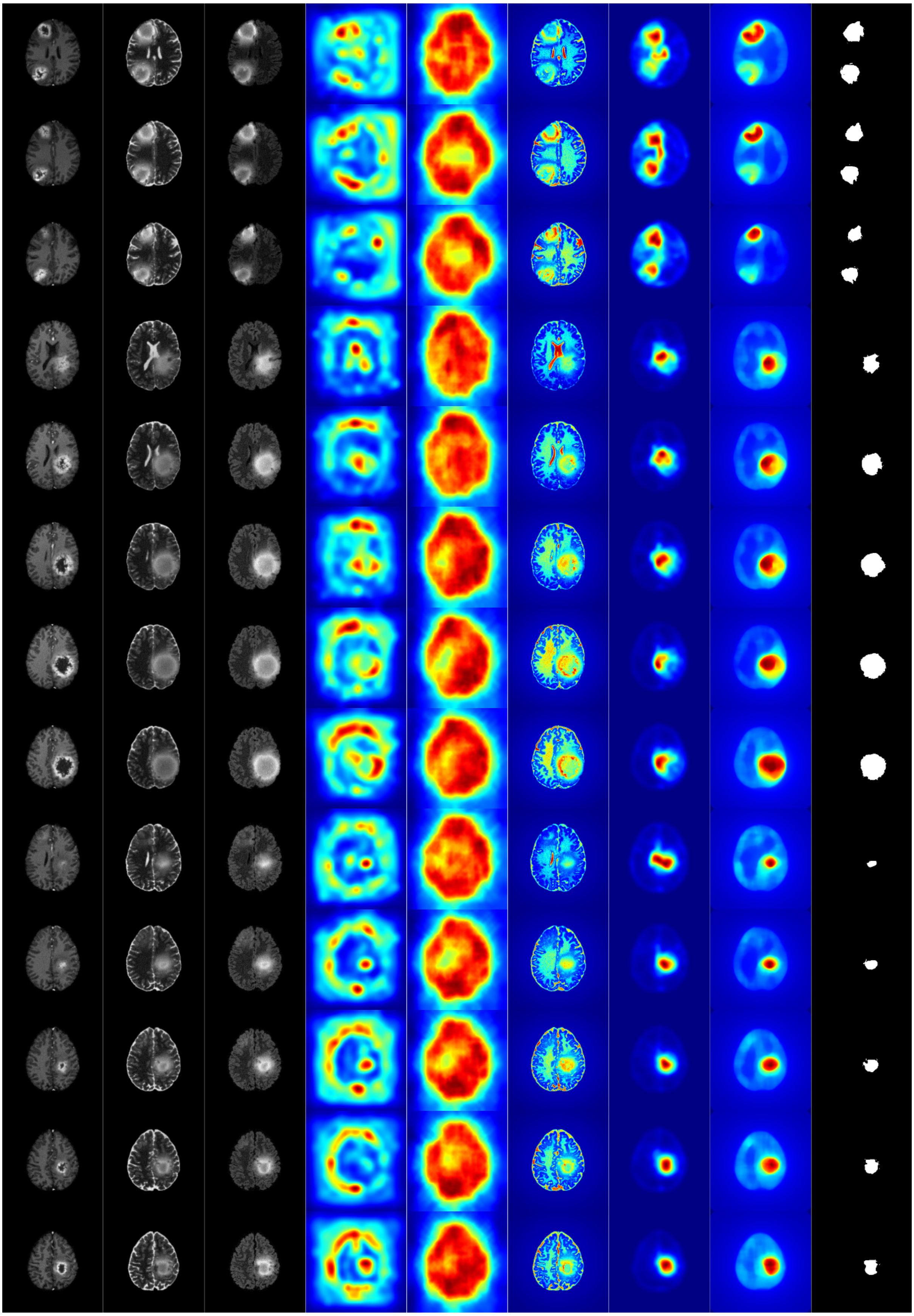

Saliency maps for 2D MR slices with SimLG GBM

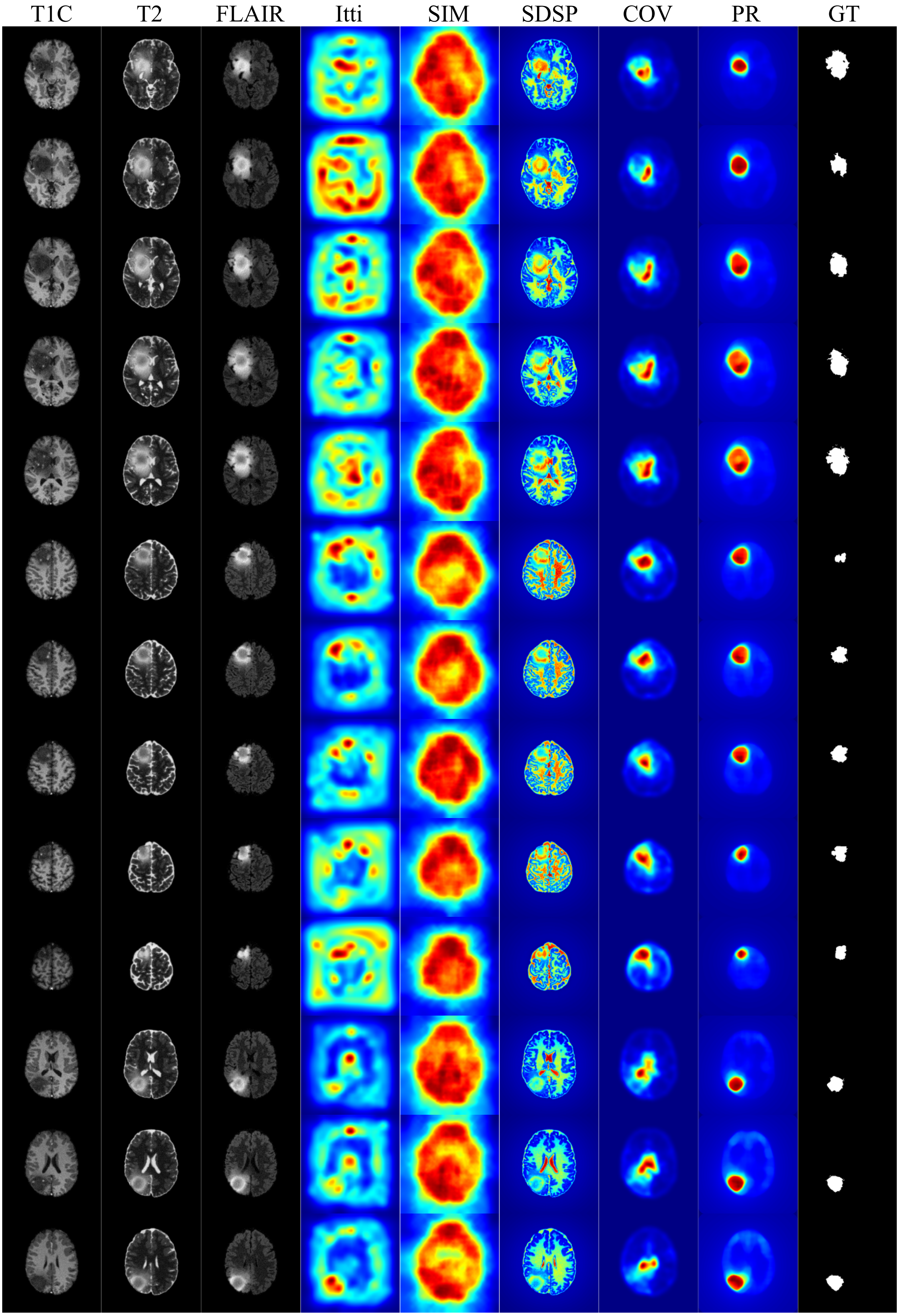

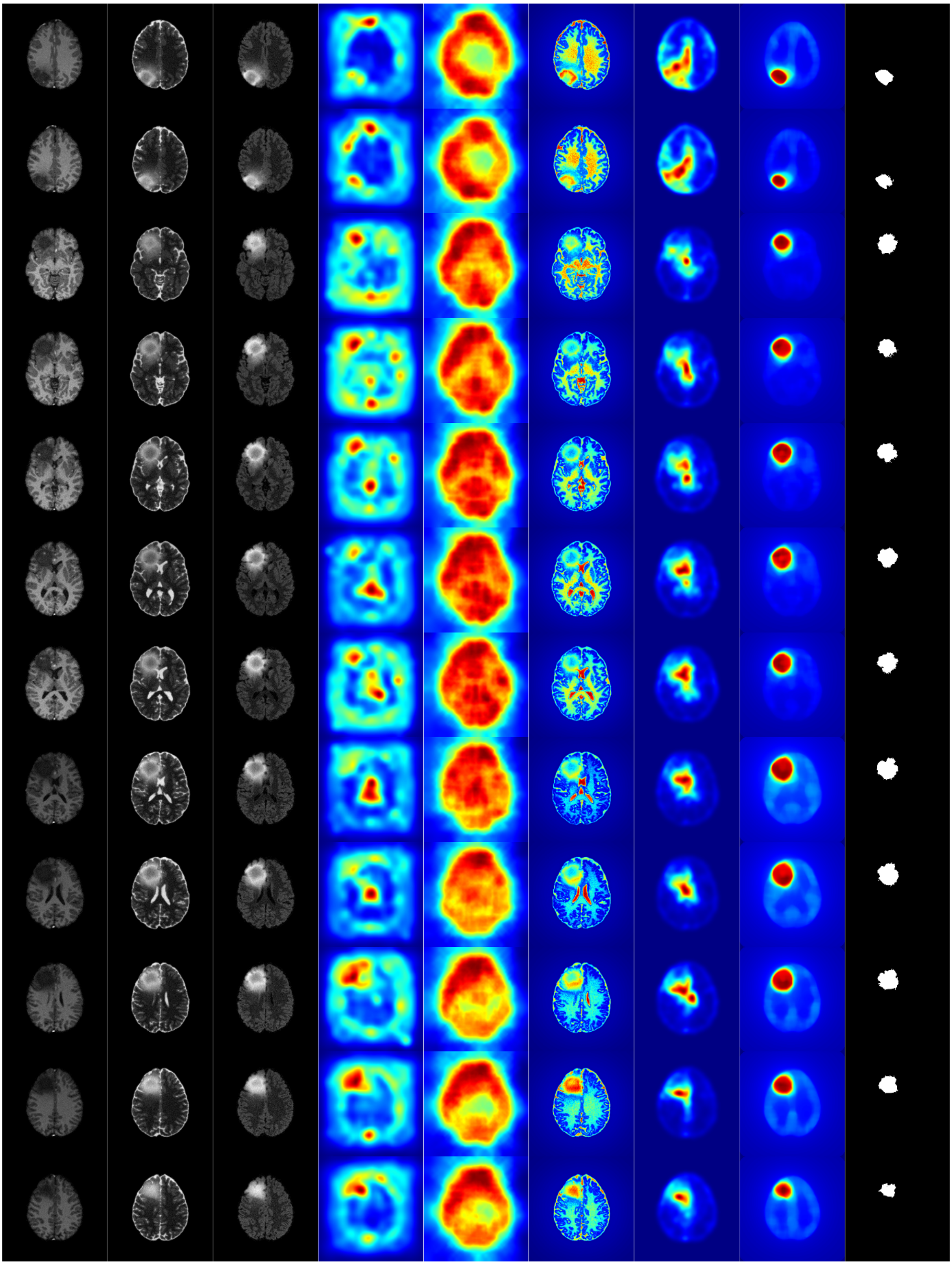

Supplement: S1 Fig — (PDF) [file pone.0146388.s001.pdf]
